# The relative importance of affect variability and mean levels of affect in predicting complex task performance

**DOI:** 10.3389/fpsyg.2024.1344350

**Published:** 2024-08-21

**Authors:** Maddison N. North, Jonathan T. Huck, Eric Anthony Day, Ashley G. Jorgensen, Kelsey A. Richels

**Affiliations:** ^1^Department of Psychology, University of Oklahoma, Norman, OK, United States; ^2^Institutional Analytics & Research, Western Governors University, Salt Lake City, UT, United States; ^3^Human Resources Research Organization (HumRRO), Alexandria, VA, United States; ^4^Colarelli, Meyer & Associates, Denver, CO, United States

**Keywords:** affect variability, relative importance, complex task performance, adaptability, off-task attention

## Abstract

Although research indicates affect variability—the extent to which an individual’s emotions fluctuate—is associated with behavioral outcomes related to adjustment and adaptability, it is unclear to what extent findings make important contributions to the literature when past research has failed to account for the role of mean levels of emotion. Accordingly, we conducted a repeated-measures laboratory study of college students (*N* = 253) learning to perform a complex computer task to examine the relative importance of affect variability indices (i.e., spin, pulse, and flux) compared to mean levels in explaining variance in off-task attention and task performance before and after changes in task demands (i.e., skill acquisition and adaptation). In doing so, we also disentangled valence and arousal (i.e., activating versus deactivating) aspects of emotion. Relative importance analyses showed mean levels of emotion were the most dominant predictors (i.e., explained the most variance)—negative deactivating emotions for off-task attention and positive activating emotions for performance. However, flux in negative activating and negative deactivating emotions also explained enough variance to be considered important, suggesting that flux has been overlooked in empirical research. Our findings also highlight that future research must account for mean levels when examining relationships between affect variability and outcomes of interest.

## 1 Introduction

Success in today’s world depends on adaptability (Pulakos et al., 2000; Baird and Griffin, 2006; Ployhart and Bliese, 2006), or the capacity to learn complex tasks and adjust to change (Bell et al., 2017). It is important to consider underlying individual differences that make individuals more or less adaptable. Non-cognitive traits like personality may contribute to the capacity to learn (i.e., skill acquisition) and adjust to change (i.e., adaptive performance), yet the literature shows relatively weak relationships between non-cognitive traits (i.e., the Big Five) and task performance (Allworth and Hesketh, 1999; Pulakos et al., 2002; Huang et al., 2014). Given the emotional nature inherent in learning new tasks and adapting to unexpected changes (Kiefer, 2002), it seems important to consider the role of emotions, or how mean levels of emotion (e.g., average ratings of positive and negative emotions from the PANAS; Watson et al., 1988) are associated with an individual’s capacity to learn and adapt. However, as a dynamic criterion, learning and adaptive performance may require a dynamic predictor beyond average emotion scores (Huang et al., 2014). The premise of the present study is that fluctuations in the expression of traits are also important to understanding human phenomena (Fleeson and Jayawickreme, 2015; Beckmann and Wood, 2017). Affect variability indices—spin, pulse, and flux (which will be defined shortly)—are traits that reflect individual differences in within-person variability (i.e., the extent to which an individual differs in how their emotions fluctuate and change over time and circumstances). Affect variability trait scores are computed via repeated measurements of emotional states, and they reflect personality constructs that are meaningfully distinct from common conceptualizations and operationalizations of personality (e.g., Big Five dimensions of personality; Moskowitz and Zuroff, 2004; Richels et al., 2020) by accounting for the dynamic nature of personality. Accordingly, they hold promise for extending theory on the role non-cognitive traits like personality play in behavioral phenomena.

Extant theory and empirical evidence suggest affect variability should explain variance in behavioral outcomes (e.g., interpersonal agentic and communal behaviors, task effort, withdrawal behaviors, organizational citizenship behaviors, counterproductive work behaviors; Timmermans et al., 2010; Chandler, 2012; Clark et al., 2018; Richels et al., 2020). Although research has begun to examine the dynamic forms of emotion and how they relate to various outcomes of interest such as venture goal progress (Uy et al., 2017) and reactions to work–family conflict (Yang and Dahm, 2021), limited research has examined dynamic emotion expression in relation to task performance. Indeed, our review of the literature revealed only three studies that examined associations between affective variability and performance scores, two of which examined self-rated performance and/or supervisor perceptions of performance (i.e., Shapiro, 2015; De Longis and Alessandri, 2020), and only one that examined objective task performance (i.e., Richels et al., 2020). Recognizing that none of this research statistically accounted for mean levels, and considering the weak relationships found between task performance and traditional personality variables (Huang et al., 2014), the purpose of our study was to examine whether affect variability could be key to explaining variance in performance beyond mean levels.

Disentangling the effects of different forms of within-person fluctuations relative to mean levels is important to developing comprehensive yet parsimonious frameworks of the non-cognitive trait determinants of behavioral outcomes. Our comprehensive empirical approach distinguishes valence and arousal (i.e., activation) aspects of emotion and contributes to theory by clarifying the relevance of affect variability to understanding task performance. In doing so we also contribute to the literature by providing methodological guidance for future research on affect variability. Per Nathans et al. (2012), we investigated the relative importance of affect variability indices and mean levels of emotions to off-task attention and task performance in the context of learning a complex computer game used in past research on self-regulation, skill acquisition, and adaptive performance (e.g., Hughes et al., 2013; Hardy et al., 2014, 2023).

## 2 Affect, attention, and task performance

Although constructs like emotion, feeling, and affect are often used interchangeably, with affect used as an umbrella term, there are important distinctions made in the research literature (Gooty et al., 2009). Emotions are discrete states of short duration that tend to be in response to an external stimulus (Cropanzano et al., 1993; Weiss and Cropanzano, 1996; Mulligan and Scherer, 2012) that reflect an individual’s affect disposition (Revelle and Scherer, 2009). Affect reflects an individual’s stable emotional disposition and is typically defined as positive (i.e., pleasant) or negative (i.e., unpleasant) in nature (Shockley et al., 2012). Emotions have been found to be important for a wide array of behavioral outcomes (e.g., interpersonal interactions, leadership behaviors, task performance; Ashkanasy and Dorris, 2017).

The role of affect has been studied extensively within the context of learning. Findings suggest state and trait affect can have important effects on the allocation of attentional resources to task demands and specific cognitive processes such as decision-making, reasoning, and memorization (Taylor, 2001; Chaffar and Frasson, 2004; Tyng et al., 2017). Research indicates that positive affect is beneficial for these cognitive processes (Chaffar and Frasson, 2004; Jorgensen et al., 2021), and is associated with greater attention allocation toward a task (Merlo et al., 2018). However, emotions experienced at high levels of intensity, positive or negative, can be detrimental to concentration, memorization, attention, and reasoning (Chaffar and Frasson, 2004; Ochs and Frasson, 2004). In these instances, emotions may pull attention away from task demands (Vuilleumier, 2005).

It is common to experience an array of emotions when learning new tasks or adapting to changes in task demands (Kiefer, 2002). Research generally suggests that positive state and trait affect positively predict and are beneficial to task performance (Fortunato and Williams, 2002; Hu and Kaplan, 2015; Fodor and Pintea, 2017; Merlo et al., 2018), whereas negative state and trait affect are inversely related to or are detrimental to task performance (Isen, 2001; Shockley et al., 2012; Lin et al., 2014; Chi et al., 2015; Graziotin, 2016; Fodor and Pintea, 2017; Jorgensen et al., 2021; Lester et al., 2022).^1^ However, it is important to note that some findings have been inconsistent with this pattern (Jefferies et al., 2008).

Research examining the relationships between affect and task performance points to the role of task attention as a key mediating mechanism as lapses in attention are detrimental to performance (Smallwood, 2011). Positive trait affect serves to activate the behavioral activation system, a physiological mechanism thought to spur task attention and goal pursuit (Carver and White, 1994), which triggers reward seeking behaviors that lead to positive performance outcomes (Fodor and Pintea, 2017). High positive trait affect is associated with framing complex tasks as growth opportunities, and greater task enjoyment, satisfaction, persistence, and achievement (Fortunato and Williams, 2002; Boehm and Lyubomirsky, 2008; Lanaj et al., 2012). In contrast, high negative trait affect is associated with viewing complex tasks as threats, and experiencing greater distress, cognitive exhaustion, and task withdrawal (Necowitz and Roznowski, 1994; Fortunato and Williams, 2002; Kaplan et al., 2009). Positive state and trait affect are beneficial for focusing attention (Fredrickson and Branigan, 2005; LeBlanc et al., 2015). In comparison, negative state and trait affect act as a distraction that must be regulated, limiting attentional resources and directing attention off-task (Smallwood et al., 2009; Rowe and Fitness, 2018).

The role of attention is particularly important to adaptive performance as individuals must focus on recognizing changes, identifying new task demands, and developing strategies to improve performance (Jundt and Shoss, 2023). Although directing attentional resources toward the task enables individuals to meet new demands (Kanfer and Ackerman, 1989; Randall et al., 2014; Jundt et al., 2015; Niessen and Jimmieson, 2016), task changes and increases in complexity can influence how well individuals maintain task attention (Jorgensen et al., 2021). Changes in a task environment can produce an emotional response that must be managed to curb attentional resources from getting pulled away from task demands (Jundt and Shoss, 2023).

The commonly used two-dimensional valence approach to examining affect-performance relationships (e.g., Chi et al., 2015) likely limits empirical research given the potential of additional dimensions. Although several other dimensions have been considered (e.g., agency, certainty, regulatory focus, and situational-control), parsing arousal (i.e., activating and deactivating emotions) from valence (i.e., positive and negative emotions) appears to hold the most promise (Smith and Ellsworth, 1985; Carroll et al., 1999; Kuppens et al., 2013; Bliss-Moreau et al., 2020). When disentangling affect in this way, state and trait affect are examined across four dimensions: positive activating (e.g., excited, happy), positive deactivating (e.g., calm, relaxed), negative activating (e.g., angry, anxious), and negative deactivating (e.g., bored, disappointed). Research demonstrates this four-dimensional approach is useful for explaining variance in various criteria such as consumer behavior (e.g., customer evaluation, buying behavior; Kranzbühler et al., 2020), cognitive processing (Delaney-Busch et al., 2016), attention, and creative task performance (Tidikis et al., 2017). In particular, research following a dual-pathway theory of affect and creative performance shows that positive activating affect spurs cognitive flexibility, while negative activating affect induces cognitive perseverance (De Dreu et al., 2008; Nijstad et al., 2010). With respect to complex task performance, positive activating emotions have been found to yield a stronger relationship with performance than positive deactivating emotions (Jorgensen et al., 2021). Individuals vary widely in their affective experience of valence and arousal such that these dimensions must be accounted for when examining the role of affect in behavioral phenomena (Kuppens et al., 2013).

## 3 Between-person differences in within-person variability

Single-occasion measurements of personality, such as with Big Five measurement, fail to address daily personality expression (Debusscher et al., 2017), which is not static but fluctuates within individuals differently (Judge et al., 2014; Fleeson and Jayawickreme, 2015). Personality-based variables are more nuanced and dynamic than what can be assessed via single-occasion measurement (Judge et al., 2014; Jayawickreme et al., 2019), which suggests that personality variables, such as positive or negative trait affect, may have important relationships with outcomes of interest not accounted for by traditional measures (Judge et al., 2014). Individuals with similar scores as measured at any single point in time may react differently to the same situation (Judge et al., 2014; Jayawickreme et al., 2019), indicating that research needs to account for between-person differences that may be responsible for this variation. Dynamic approaches are needed (Minbashian et al., 2010). Individuals with identical mean-level scores will not necessarily have identical scores for within-person variability (fluctuations). Indeed, a burgeoning empirical literature shows that within-person variability in non-cognitive traits predicts job performance (Beckmann et al., 2020) and provides incremental validity in explaining outcomes, such as adaptive performance, above scores derived from traditional measures of non-cognitive traits (Minbashian et al., 2010).

Personality theorists increasingly emphasize the need to account for consistency and variability, recognizing that although an individual’s average behavior can be consistent, their behavior can vary across contexts (Fleeson and Jayawickreme, 2015; Jayawickreme et al., 2019). Fleeson and Jayawickreme (2015) conceptualize personality traits as a density distribution of states and suggest there are between-person differences in individual means *and* variability. In this way, over the last 25 years researchers have espoused the importance of capturing individual differences in affect variability through a variety of indices (e.g., spin, pulse, and flux), which are terms used to refer to the trait-like aspects of personality that can leverage emotion scores to describe between-person differences in within-person variability (Clark et al., 2018; Chester et al., 2020; Richels et al., 2020). To the extent that behavior reflects *dynamic* personality factors such as affect variability, research needs to disentangle mean levels from within-person variability when examining personality-outcome relationships (Judge et al., 2014), particularly when considering the weak relationships found between traditional measures of personality and performance (Huang et al., 2014).

## 4 Affect variability

Affect variability is a stable trait that reflects variability in affect, is conceptually different from mean levels of affect, and holds considerable potential as a meaningfully distinct aspect of personality that captures differences in how people react emotionally across a range of situations, performance contexts, and life events (Eid and Diener, 1999; Kuppens et al., 2007; Beal et al., 2013; Richels et al., 2020). An individual’s emotional disposition or general affect can be depicted as residing on a circumplex of pleasure (pleasant-unpleasant) and arousal (activated-deactivated). Affect variability is conceptualized as the ways in which an individual’s affect moves around this circumplex, reflecting how their affect may fluctuate across time. This movement differs across individuals as they vary in their experience of more or less pleasant/unpleasant and activated/deactivated states (Kuppens et al., 2007). Beyond scores taken at one point in time or mean levels derived from multiple administrations, scholars have made the case that it is critical to examine and understand the within-person variability in scores taken across multiple time points (Fleeson, 2001; Kopala-Sibley et al., 2013). In this way, affect variability scores capture differences in the dynamic experience of emotions, and thus may improve the variance explained in dynamic behavior and outcomes beyond traditional personality scores, even scores for emotional stability (Richels et al., 2020).

With respect to complex task performance, the role of affective variability can be understood in terms of stress-attention-performance relationships. Individuals with high affect variability tend to react with greater intensity to emotional events (Beal et al., 2013), have lower subjective wellbeing (Kuppens et al., 2007; Uy et al., 2017), and have more difficulty adjusting to change, even when accounting for trait negative affect (Beal and Ghandour, 2011). Changes in task demands or increases in complexity can cause an emotional response that diverts attention and impedes task performance, particularly adaptive performance (Niessen and Jimmieson, 2016; Jundt and Shoss, 2023). The need to regulate one’s response may be distracting and deplete cognitive resources, lowering the individual’s ability to understand task demands and respond accordingly (Richels et al., 2020). Those high in affect variability are more likely to view complex, dynamic task environments as distressing, leading to self-doubt, worry, and mind wandering, all of which are aspects of off-task attention and undermine performance (Gopher et al., 2000; Bailey and Konstan, 2006; Randall et al., 2014). There has been a call for research to examine the relationship between affect variability indices and off-task attention, as off-task attention has been proposed to function as a key explanatory mechanism linking indices of affect variability to task performance (Richels et al., 2020). Indeed, research has found that fluctuations in affect can influence performance through their effects on attention allocation. Specifically, fluctuations in negative affect are related to reduced on-task attention, which is harmful for performance (Merlo et al., 2018). Although the relationships between attention and dimensions of emotional valence and arousal have been studied (Jefferies et al., 2008), the relationship between affect variability and off-task attention has not been examined directly.

### 4.1 Indices of affect variability: spin, pulse, and flux

The potential in affect variability as an important component of personality lies in its different conceptualizations and operationalizations. The scholarly literature points to several different but related aspects of affective variability, namely spin, pulse, and flux. Each describes how an individual’s emotions move around the affect circumplex (Kuppens et al., 2007). The circumplex structure is robust and can be leveraged to represent individual differences in emotional experiences, which vary across individuals (Barrett and Bliss-Moreau, 2009). Affect spin refers to an individual’s variability in distinct emotions across the full range of dimensions—valence and activation—such that higher spin indicates greater fluctuations across the full circumplex. Higher spin scores would suggest that an individual experiences a greater array of emotions over time, whereas lower spin scores would indicate that an individual tends to experience similar emotions over time (Kuppens et al., 2007). Affect pulse refers to fluctuations in intensity regardless of valence and activation, such that higher pulse reflects a greater mix of low and high intensity in emotions over time and events (Moskowitz and Zuroff, 2004; Kuppens et al., 2007). Individuals with lower pulse scores tend to feel emotions at a similar level of intensity, strongly or weakly, regardless of the emotion. In comparison, individuals with higher pulse scores experience emotions at varying intensities over time (Kuppens et al., 2007). Flux refers to fluctuations (i.e., within-person *SD*s) along specific dimensions, whether valence or activation, or a combination, comprising the four quadrants of the affect space: positive activating (PA), positive deactivating (PD), negative activating (NA), and negative deactivating (ND) (Kuppens et al., 2007). Higher (lower) flux scores suggest greater (lesser) variability experienced around one’s average within a particular dimension (Moskowitz and Zuroff, 2004). Flux in one space does not determine flux in another space (Chester et al., 2020). Flux thus reflects the variability around an individual’s mean level (Hardy and Segerstrom, 2017). Examining affect variability via a four-quadrant flux approach could provide nuance and theoretical value beyond spin and pulse, which may be too coarse to explain differences in the effect of affect fluctuations across individuals. For instance, it is possible for individuals to have similar levels of pulse but differences in intensity across the circumplex. Likewise, two individuals can have identical spin scores but differ with respect to which particular quadrants of the circumplex comprise their scores. In contrast to how spin and pulse cover the circumplex via a single index, multiple flux indices could be used together to cover the circumplex and still represent important distinctions in how PA, PD, NA, and ND affect function (Dolcos et al., 2004; Barrett and Bliss-Moreau, 2009).

Although early research focused on spin and pulse (Moskowitz and Zuroff, 2004; Kuppens et al., 2007), interest in flux has recently emerged (Timmermans et al., 2010; Chandler, 2012; Chester et al., 2020). Whereas some researchers suggest that spin is best suited for explaining variance in behavioral outcomes (e.g., Chandler, 2012), others have argued that all indices are worthwhile to examine (e.g., Chester et al., 2020). Recent findings have shown how spin and pulse both undermine effort and complex skill learning (Richels et al., 2020). The extant empirical literature is limited in studies that comparatively examine spin, pulse, and flux. In our literature search we only found two studies that examined all three indices (i.e., Russell et al., 2007; Chandler, 2012).With respect to flux these studies only examined positive and negative valence dimensions without distinguishing activation potential. Indeed, scholars have recognized the limitation of not considering activation in studies of flux (Chester et al., 2020). Thus, the present study makes an important contribution to the literature by comparatively examining spin, pulse, and four dimensions of flux that disentangle valence and activation (i.e., PA, PD, NA, and ND). By disentangling the valence and activation dimensions in flux scores, the present study more carefully examines the validity of spin and pulse as single indices of affective variability versus the validity of a combination of flux indices.

### 4.2 Effects beyond mean emotion levels

Affect variability scores yield stable between-person differences and are distinct from mean-level scores (Eid and Diener, 1999), yet research should still account for affect mean levels due to the unclear relationships that may exist between mean levels, variability in affect, and outcomes of interest (Russell et al., 2007). Our review of the literature found mixed results whether researchers noted mean levels as an important predictor or accounted for mean levels in their analyses. Examining mean levels in addition to affect variability is not yet a routine practice and may call into question the conclusions of past research that did not account for the effects of mean levels. Research that did not include mean levels has examined affect variability in relation to a wide range of outcomes, including fatigue and strain (Beal et al., 2013), aggressive behavior (Chester et al., 2020), career decision-making self-efficacy and career choice anxiety (Jung et al., 2015), career indecision and maturity (Park, 2015), and effort and complex skill learning (Richels et al., 2020). Research that has included mean levels has shown that affect variability can explain additional variance in a variety of health-related outcomes (e.g., sensitivity to affective events, psychological distress, physical health, alcohol consumption, partner quarrelsome behavior) beyond mean levels (Beal and Ghandour, 2011; Mohr et al., 2015; Sadikaj et al., 2015; Hardy and Segerstrom, 2017). Mean levels of positive and negative affect were still predictive and sometimes acted as stronger predictors than affect variability (e.g., Hardy and Segerstrom, 2017). Although affect variability may act as a significant predictor, distinct from mean levels, the comparative strength of these variables is not clear.

## 5 Present research

Although theoretical backing suggests affect variability is related to task performance (Chandler, 2012) and may undermine performance via its association with attention (e.g., stress-attention-performance relationship), it is critical to first establish what associations exist between affect variability, performance, and off-task attention while accounting for mean levels. To examine these relationships, participants were tasked with learning how to play a first-person shooter computer game over a series of 14 sessions while completing self-reports of off-task attention and emotions between sessions. We increased game complexity halfway through the sessions to examine the relative importance of affect variability indices and mean levels of emotions to explaining variance in off-task attention and performance in periods of acquisition (pre-task change) and adaptation (post-task change). Specifically, and following the recommendations of Nathans et al. (2012) and Nimon and Oswald (2013), we conducted a relative importance analysis of spin, pulse, and all flux indices alongside mean scores in explaining variance in off-task attention and task performance during periods of skill acquisition and adaptation. In this way, this study makes a unique contribution to understanding how affective aspects of personality relate to task performance.

Covariation among different dimensions of affect and indices of affect variability should be expected because they all (a) conceptually speak to the experience of emotions and (b) are operationally calculated using the same set of scores. In general, we expected negative (positive) mean levels would be positively (inversely) related to off-task attention and positive (negative) mean levels would be positively (inversely) related to task performance (Jorgensen et al., 2021). However, without properly accounting for covariation, it is difficult to determine which mean dimension scores or affect variability index, or subset of dimensions and indices, better or best explains variance in outcomes. Moreover, accounting for covariation is also important to understanding potential suppression effects, or when the inclusion of one predictor removes construct irrelevance from another predictor, leading to stronger estimates (Dalal et al., 2012). Accordingly, relative importance analysis involving a combination of relative importance measures (Nathans et al., 2012) is needed to inform theory on how affective variability alongside mean scores explains variance in outcomes of interest. There may be some aspects of affect variability that are not directly related to an outcome, but accounting for their covariance leads to better estimates for mean scores and other affect variability indices. Relative importance analysis can also elucidate whether any variable is completely dominant in their contribution—explaining more unique variance in an outcome regardless of which other predictors are included in a regression model (Azen and Budescu, 2003). In this way, relative importance analysis can provide a detailed yet comprehensive examination of the contributions played by affect variability in relation to mean-level scores across the affect circumplex. Our research questions compare the relationships or relative predictive strength of these variables and address whether mean level scores for any dimension or any index of affect variability show complete dominance (i.e., consistently explains the most unique variance; Nimon and Oswald, 2013) in explaining variance in off-task attention and performance.

*Research question 1:* What is the relative importance of affective variability indices compared to mean levels of affect dimensions in explaining variance in (a) off-task attention and (b) performance in acquisition (pre-task change) and adaptation (post-task change)?

*Research question 2:* Do any indices of affect variability or mean levels of affect dimensions show complete dominance in explaining variance in (a) off-task attention or (b) performance in acquisition (pre-task change) and adaptation (post-task change)?

## 6 Methods

### 6.1 Participants

Data were collected from 288 undergraduate students in the psychology participant pool from a large, public university in the southwestern United States. To receive research credit and potential entry into a gift card drawing, participants were told they would be learning a first-person-shooter computer game. Participants had to be 18 or older and proficient in English. Data were excluded for 12 participants who experienced technical difficulties and six who failed to follow instructions. An additional 17 participants were removed for careless responding, which was detected via long string analysis (Meade and Craig, 2012). Two hundred and fifty three students comprised the final sample, 85 of which were female; 169 self-reported as White or Caucasian (66.8%), 25 Asian (9.9%), 16 Hispanic/Latino (6.3%), 14 Black or African American (5.5%), 11 Multiple (two or more ethnicities) (4.4%), eight Native American (3.2%), and four Middle Eastern (1.6%). The age range was 18–30 years (*M* = 19.00, *SD* = 1.55).

### 6.2 Criterion task

Unreal Tournament 2004 (UT2004; Epic Games, 2004), a commercially available first-person shooter computer game used in prior research on self-regulation and complex task performance (e.g., Hughes et al., 2013; Hardy et al., 2014), was the performance task used in this study. The cognitive and psychomotor demands of UT2004 are fast-paced and dynamic. In addition, its technology-mediated and dynamic task qualities are representative of many simulation-based training contexts that are increasingly in use in both the private and public sectors (Hardy et al., 2019).

In UT2004, participants control their own bot using both mouse and keyboard to compete against game-controlled opponents (i.e., bots). During battle, participants can build their bot’s health, offensive capabilities, or defensive capabilities by searching for resources (e.g., armor) and new weapons, each of which has its own advantages and disadvantages. If destroyed, the participant’s bot respawns in a random location without the weapons and resources they previously found or built up. The game-controlled bots competed against the participant’s bot, as well as each other. Players must be able to adapt their tactics to a wide variety of circumstances to achieve high scores.

### 6.3 Procedure

All procedures were reviewed and approved by the University of Oklahoma Institute Review Board (#5948). Up to six participants could participate at a time, each sitting at an individual computer station. Participants completed an informed consent form and several self-report individual difference measures. A performance-based lottery was utilized as incentive. Participants were informed that for every game in which they scored in the top half of all participants, they would receive an entry to win one of five gift cards worth $25. Participants next watched a 15-min training video to learn about UT2004’s rules, basic controls, and resources. Then participants engaged in a 1-min practice game with no competing bots to help them better understand the game display, environment, and controls.

Participants completed twenty-eight 4-min games (i.e., trials). After each pair of games, they completed state-based self-report measures of emotions and attention. Performance was collapsed into 14 measurement sessions (i.e., mean performance for each pair of two games). Sessions 1–7 were pre-change, acquisition sessions. Sessions 8–14 were post-change, adaptation sessions. There were two 4-min breaks after Sessions 3 and 10 to help combat fatigue. Through Sessions 1–7, participants competed against two bots set to a difficulty level of 5 on a 1-to-8 scale, which has been established previously as an appropriate level of moderate difficulty (Hughes et al., 2013; Hardy et al., 2014). After the halfway point (i.e., after Session 7), several task demands changed without warning, designed to prompt reactive adaptation due to increasing task complexity (Hughes et al., 2013). Participants competed against nine bots set to a higher difficulty level (i.e., 6) in a new and substantially larger game environment (i.e., the game map), with more open spaces, stories of platforms, and ledges from which the player bot could fall and be destroyed. After the 14th session participants were debriefed.

### 6.4 Measures

#### 6.4.1 Mean levels and affect variability

To calculate mean levels, spin, pulse, and flux, we used an adapted version of the Positive and Negative Affect Schedule (PANAS; Watson et al., 1988). After two trials of gameplay, participants rated 16 emotions that varied in arousal/activation and valence in terms of how they felt during the past two games. Negative deactivating (ND) emotions were assessed with the adjectives discouraged, disappointed, fatigued, and bored. Negative activating (NA) emotions were assessed with words such as uneasy, frustrated, irritated, tense, anxious, and angry. Positive deactivating (PD) emotions were assessed with the words ease, calm, and relaxed. Positive activating (PA) emotions were described with the words enthusiastic, excited, and happy. Participants responded to each word on a 9-point Likert scale (1 *= very slight/not at all,* 3 *= a little,* 5 *= moderately,* 7 *= quite a bit,* 9 *= extremely*).

##### 6.4.1.1 Mean levels

Participant ratings for PA, PD, NA, and ND emotions were averaged, respectively, across the 14 sessions.

Before calculating spin, pulse, and flux, each participant’s activation and valence scores must be calculated across the 14 sessions. Per Kuppens et al. (2007), activation was scored as (PA + NA) − (PD + ND), and valence was scored as (PA + PD) − (NA + ND). Next, we calculated means and standard deviations for activation and valence. Activation variability was scored using within-person standard deviations of activation/deactivation scores, and valence variability was scored using within-person standard deviations of pleasure/displeasure scores.

##### 6.4.1.2 Affect spin

Spin was operationalized per Moskowitz and Zuroff (2004) and Kuppens et al. (2007). First, the unit vector was calculated for each session:

$\left(\frac{\mathrm{valenc}e_{t}}{\sqrt{\mathrm{valenc}e_{t}^{2}+\mathrm{activatio}n_{t}^{2}}},\frac{\mathrm{activatio}n_{t}}{\sqrt{\mathrm{valenc}e_{t}^{2}+\mathrm{activatio}n_{t}^{2}}}\right)$

Next, for each participant, we calculated *R*, the vector of all observations:

$\left(\sum_{t=1}^{n}\frac{\mathrm{valenc}e_{t}}{\sqrt{\mathrm{valenc}e_{t}^{2}+\mathrm{activatio}n_{t}^{2}}},\overset{n}{\underset{t=1}{\sum }}\frac{\mathrm{activatio}n_{t}}{\sqrt{\mathrm{valenc}e_{t}^{2}+\mathrm{activatio}n_{t}^{2}}}\right)$

Next, we calculated the length of *R*:

$\sqrt{\frac{\sum_{t=1}^{n}\frac{\mathrm{valenc}e_{t}}{\sqrt{\mathrm{valenc}e_{t}^{2}+\mathrm{activatio}n_{t}^{2}}}+\sum_{t=1}^{n}\frac{\mathrm{activatio}n_{t}}{\sqrt{\mathrm{valenc}e_{t}^{2}+\mathrm{activatio}n_{t}^{2}}}}{n}}$

Depending on the variability in the angles, the *R*’s length $\left(\frac{\left\|\vec{R}\right\|}{n}\right)$ can range from 0 to 1. There is no variability if $\left(\frac{\left\|\vec{R}\right\|}{n}\right)$ is equal to 1. $\left(\frac{\left\|\vec{R}\right\|}{n}\right)$ approaches 0 as the angles become more dispersed, due to them canceling each other out (Kuppens et al., 2007). In the last step, the standard deviation of the angles of the unit vectors provides a value for spin:

$\sqrt{-2\ln\left(\frac{\left\|\vec{R}\right\|}{n}\right)}$

Scores range from 0 to infinity (Kuppens et al., 2007).

##### 6.4.1.3 Affect pulse

Calculations per Moskowitz and Zuroff (2004) and Kuppens et al. (2007) were also used to operationalize pulse:

$\sqrt{\mathrm{valenc}e_{t}^{2}+\mathrm{activatio}n_{t}^{2}}$

##### 6.4.1.4 Affect flux

Flux was scored for each emotion dimension (PA, PD, NA, ND) using the within-person standard deviation across all sessions (Moskowitz and Zuroff, 2004).

#### 6.4.2 Off-task attention

We adapted items from Kanfer et al. (1994) to create a 5-item measure of off-task attention (e.g., “I lost interest in Unreal Tournament for short periods” and “I took ‘mental breaks’ during Unreal Tournament”). After each session, participants responded in reference to the past two games using a 7-point Likert scale (1 = *never*, 7 = *constantly*). The mean coefficient alpha reliability estimates across all 14 sessions was 0.90.

#### 6.4.3 Task performance

Task performance scores were calculated by taking the number of kills (i.e., times the participant bot destroyed a computer bot), divided by the quantity of kills plus deaths (i.e., times the participant bot was destroyed), plus participant rank relative to the bots, multiplied by 100 for each game trial (Hardy et al., 2014, 2019). Scores were averaged across game pairs to index session scores.

## 7 Results

### 7.1 Relative importance analysis

Table 1 shows the descriptive statistics, zero-order correlations, and average off-task attention and performance scores across sessions. The direction of significant correlations between mean levels and the outcomes of interest aligned with our expectations. Growth curve analysis per procedures recommended by Bliese and Lang (2016) demonstrated off-task attention scores increased at a linear rate across sessions [*t*(3288) = 9.11, *B* = 0.07, *p* < 0.01]: Session 1 *M* = 1.89, *SD* = 1.09; Session 14 *M* = 2.86, *SD* = 1.92. Performance scores showed a classic learning curve in acquisition with significant positive linear [*t*(3284) = 14.19, *B* = 5.28, *p* < 0.01] and negative quadratic effects [*t*(3284) = −9.14, *B* = −0.54, *p* < 0.01], followed by a sharp discontinuous drop [*t*(3284) = −21.79, *B* = −18.45, *p* < 0.01] and a slower rate of reacquisition following the task change [*t*(3284) = −8.09, *B* = −4.18, *p* < 0.01]: Session 1 *M* = 27.67, *SD* = 18.13; Session 7 *M* = 41.60, *SD* = 21.67; Session 8 *M* = 27.87, *SD* = 15.60; Session 14 *M* = 31.44, *SD* = 19.64.

**Table 1:** Descriptive statistics and correlations.

Variable	*M*	*SD*	1	2	3	4	5	6	7	8	9	10	11	12	13	14
1. Spin	1.25	0.40														
2. Pulse	2.22	0.97	0.13*													
3. ND flux	1.13	0.56	0.41**	0.62**												
4. NA flux	1.08	0.62	0.47**	0.59**	0.65**											
5. PD flux	1.25	0.61	0.38**	0.46**	0.31**	0.53**										
6. PA flux	1.28	0.62	0.46**	0.48**	0.51**	0.47**	0.53**									
7. Mean ND	3.80	1.59	0.11	0.35**	0.55**	0.43**	0.08	0.15*	(0.96)							
8. Mean NA	3.44	1.68	0.14*	0.36**	0.49**	0.49**	0.01	0.13*	0.78**	(0.96)						
9. Mean PD	4.11	1.77	−0.13*	−0.10	−0.25**	−0.23**	0.18**	0.02	−0.34**	−0.52**	(0.96)					
10. Mean PA	3.78	1.80	−0.07	−0.17**	−0.28**	−0.21**	0.00	−0.01	−0.52**	−0.35**	0.52**	(0.96)				
11. Pre-change OTA	2.15	1.10	0.08	0.17**	0.34**	0.20**	0.06	−0.02	0.55**	0.39**	−0.23**	−0.38**	(0.95)			
12. Post-change OTA	2.66	1.44	0.06	0.22**	0.41**	0.31**	0.12	0.05	0.59**	0.40**	−0.25**	−0.45**	0.78**	(0.96)		
13. Pre-change performance	37.51	18.48	−0.05	−0.18**	−0.21**	−0.28**	0.00	−0.02	−0.48**	−0.47**	0.34**	0.50**	−0.44**	−0.36**	(0.97)	
14. Post-change performance	30.11	16.45	−0.08	−0.17**	−0.26**	−0.27**	0.00	−0.06	−0.50**	−0.45**	0.35**	0.53**	−0.46**	−0.48**	0.88**	(0.96)

ND, negative deactivating; NA, negative activating; PD, positive deactivating; PA, positive activating; OTA, off-task attention. Pre-task change off-task attention and performance are averages across the first seven sessions. Post-task change off-task attention and performance are averages across the last seven sessions. Coefficient alpha reliabilities are on the diagonal. *N* = 253. *p < 0.05, **p < 0.01, two-tailed.

We conducted a relative importance analysis per Nimon and Oswald (2013). We used a relative weight importance (RWI, or variance explained in the outcome scores) of 0.02 (i.e., 2%) as our *a priori* standard for determining if a variable was an “important” predictor. Dominance was determined by taking each pair of predictors and comparing their contributions across all models including all combinations of all predictor variables (Azen and Budescu, 2003; Luchman, 2015). We focused on which (if any) established complete dominance, denoting a certain predictor explained the highest amount of variance across all subsets of predictors (Azen and Budescu, 2003).

Although not part of our main investigation, we also looked for the presence of suppression effects by examining how unique variance contributions changed given model size. Typically, as a model’s size increases (with added predictors) the unique variance explained by a single predictor will decrease as the variance is shared among more predictors (Azen and Budescu, 2003; Nathans et al., 2012). However, a suppressor will work in the opposite way by explaining little variance on its own but explaining more variance overall as the number of predictors increases.

#### 7.1.1 Off-task attention

##### 7.1.1.1 Research question 1: relative importance of affect variability

A comparison across all relative-weight analysis statistics for off-task attention during acquisition and adaptation can be found in Table 2. Predictors that met our *a priori* threshold (RWI = 0.02) in pre-change include mean ND (RWI = 0.147, RWI% = 41.88), mean PA (RWI = 0.058, RWI% = 16.52), mean NA (RWI = 0.051, RWI% = 14.53), and ND flux (RWI = 0.044, RWI% = 12.54). These variables were also above threshold in post-change: mean ND (RWI = 0.154, RWI% = 37.02), mean PA (RWI = 0.086, RWI% = 20.67), mean NA (RWI = 0.047, RWI% = 11.30), and ND flux (RWI = 0.059, RWI% = 14.18). NA flux (RWI = 0.023, RWI% = 5.53) was also found to be above threshold in post-change. There was not a clear difference in activation potential, but aside from mean PA, all predictors that met threshold had a negative valence. Although negative flux variables demonstrated having importance for predicting off-task attention, with ND flux being commensurate to mean PA and mean NA, the relative importance of the affect variability indices was generally weaker than that of mean levels. It is interesting to note that while prior literature has focused on the effects of spin and pulse, these indices did not meet threshold and were among the weakest predictors for off-task attention, both in acquisition and adaptation.

**Table 2:** Summary of statistics determining independent variable contributions to regression effects for off-task attention.

Variable	*β*	RWI	RWI%
Pre-change			
Spin	0.040	0.003	0.85
Pulse	−0.026	0.008	2.28
ND flux	0.191	0.044	12.54
NA flux	−0.121	0.009	2.56
PD flux	0.140	0.005	1.42
PA flux	−0.204	0.013	3.70
Mean ND	0.511	0.147	41.88
Mean NA	−0.066	0.051	14.53
Mean PD	−0.036	0.013	3.70
Mean PA	−0.098	0.058	16.52
Total	N/A	0.351	100
Post-change			
Spin	−0.087	0.004	0.96
Pulse	−0.100	0.010	2.40
ND flux	0.230	0.059	14.18
NA flux	0.070	0.023	5.53
PD flux	0.138	0.010	2.40
PA flux	−0.145	0.008	1.92
Mean ND	0.507	0.154	37.02
Mean NA	−0.149	0.047	11.30
Mean PD	−0.035	0.015	3.61
Mean PA	−0.165	0.086	20.67
Total	N/A	0.416	100

*β*, standardized regression coefficient in the full model; RWI, relative weight importance; % of variance explained in y by the given index; RWI%, % of variance explained in y by all the indices attributed to the given index. Pre-change refers to off-task attention in the first seven sessions. Post-change refers to off-task attention in the last seven sessions. ND, negative deactivating; NA, negative activating; PD, positive deactivating; PA, positive activating. *N* = 253.

##### 7.1.1.2 Research question 2: dominance

When determining if affect variability or mean levels showed complete dominance in explaining variance in off-task attention, findings revealed that mean ND was the strongest direct predictor of off-task attention across multiple indices. In addition to yielding the largest beta weight (acquisition: *β* = 0.51, *p* < 0.001; adaptation: *β* = 0.50, *p* < 0.001) in the full model. Dominance analysis across acquisition and adaptation (Table 3) confirm complete dominance for mean ND over all other variables, as it explained more unique variance in the regression effect than the other indices across all multiple regression sub-models that included that variable.

**Table 3:** Paired dominance metrics for off-task attention.

	Pre-change off-task attention			Post-change off-task attention		
	Complete	Conditional	General	Complete	Conditional	General
Spin > Pulse	0.5	0.5	0.0	0.5	0.5	0.0
Spin > ND flux	0.0	0.0	0.0	0.0	0.0	0.0
Spin > NA flux	0.5	0.0	0.0	0.5	0.5	0.0
Spin > PD flux	0.5	0.5	0.0	0.5	0.0	0.0
Spin > PA flux	0.5	0.5	0.0	0.5	0.5	0.0
Spin > Mean ND	**0.0**	**0.0**	**0.0**	**0.0**	**0.0**	**0.0**
Spin > Mean NA	0.5	0.0	0.0	0.5	0.0	0.0
Spin > Mean PD	0.5	0.5	0.0	0.5	0.5	0.0
Spin > Mean PA	0.5	0.0	0.0	0.0	0.0	0.0
Pulse > ND flux	0.5	0.0	0.0	0.0	0.0	0.0
Pulse > NA flux	0.5	0.0	0.0	0.5	0.5	0.0
Pulse > PD flux	0.5	0.5	1.0	0.5	0.5	1.0
Pulse > PA flux	0.5	0.5	0.0	0.5	0.5	1.0
Pulse > Mean ND	**0.0**	**0.0**	**0.0**	**0.0**	**0.0**	**0.0**
Pulse > Mean NA	0.5	0.0	0.0	0.5	0.0	0.0
Pulse > Mean PD	0.5	0.0	0.0	0.5	0.5	0.0
Pulse > Mean PA	0.0	0.0	0.0	0.0	0.0	0.0
ND flux > Mean ND	**0.0**	**0.0**	**0.0**	**0.0**	**0.0**	**0.0**
ND flux > Mean NA	0.5	0.5	0.0	0.5	1.0	1.0
ND flux > Mean PD	0.5	1.0	1.0	0.5	1.0	1.0
ND flux > Mean PA	0.5	0.5	0.0	0.5	0.5	0.0
NA flux > ND flux	0.5	0.0	0.0	0.0	0.0	0.0
NA flux > Mean ND	**0.0**	**0.0**	**0.0**	**0.0**	**0.0**	**0.0**
NA flux > Mean NA	0.5	0.5	0.0	0.5	0.0	0.0
NA flux > Mean PD	0.5	0.5	0.0	0.5	1.0	1.0
NA flux > Mean PA	0.5	0.5	0.0	0.0	0.0	0.0
PD flux > Mean ND	**0.0**	**0.0**	**0.0**	**0.0**	**0.0**	**0.0**
PD flux > Mean NA	0.5	0.5	0.0	0.5	0.5	0.0
PD flux > Mean PD	0.5	0.5	0.0	0.5	0.5	0.0
PD flux > Mean PA	0.5	0.5	0.0	0.5	0.0	0.0
PD flux > ND flux	0.0	0.0	0.0	0.0	0.0	0.0
PD flux > NA flux	0.5	0.5	0.0	0.5	0.5	0.0
PA flux > Mean ND	**0.0**	**0.0**	**0.0**	**0.0**	**0.0**	**0.0**
PA flux > Mean NA	0.5	0.5	0.0	0.5	0.5	0.0
PA flux > Mean PD	0.5	0.5	1.0	0.5	0.5	0.0
PA flux > Mean PA	0.5	0.5	0.0	0.5	0.0	0.0
PA flux > ND flux	0.5	0.5	0.0	0.5	0.0	0.0
PA flux > NA flux	0.5	0.5	1.0	0.5	0.5	0.0
PA flux > PD flux	0.5	0.5	1.0	0.5	0.5	1.0
Mean NA > Mean ND	**0.0**	**0.0**	**0.0**	**0.0**	**0.0**	**0.0**
Mean PD > Mean ND	**0.0**	**0.0**	**0.0**	**0.0**	**0.0**	**0.0**
Mean PD > Mean NA	0.5	0.0	0.0	0.0	0.0	0.0
Mean PA > Mean ND	**0.0**	**0.0**	**0.0**	**0.0**	**0.0**	**0.0**
Mean PA > Mean NA	0.5	0.5	1.0	1.0	1.0	1.0
Mean PA > Mean PD	1.0	1.0	1.0	1.0	1.0	1.0

Pre-change refers to the first seven sessions. Post-change refers to the last seven sessions. ND, negative deactivating; NA, negative activating; PD, positive deactivating; PA, positive activating; 1.0, first listed variable is dominant; 0.0, second listed variable is dominant; 0.5, dominance cannot be determined. Bolded font highlights the results for the variable yielding complete dominance overall. *N* = 253.

#### 7.1.2 Performance

##### 7.1.2.1 Research question 1: relative importance of affect variability

A relative importance comparison across all statistics for performance during acquisition and adaptation can be found in Table 4. Variables that met our *a priori* threshold in acquisition include mean PA (RWI = 0.131, RWI% = 36.39), mean NA (RWI = 0.076, RWI% = 21.11), mean ND (RWI = 0.073, RWI% = 20.28), mean PD (RWI = 0.034, RWI% = 9.44), and NA flux (RWI = 0.024, RWI% = 6.67). Similarly, in adaptation these same predictors met threshold: mean PA (RWI = 0.153, RWI% = 40.91), mean ND (RWI = 0.083, RWI% = 22.19), mean NA (RWI = 0.062, RWI% = 16.58), mean PD (RWI = 0.034, RWI% = 9.09), and NA flux (RWI = 0.020, RWI% = 5.35). All mean level variables were found to be important for predicting performance in pre- and post-task change. The relative weights of mean levels were larger than the relative weights of the affect variability indices, with NA flux as the only index of affect variability to reach threshold. NA flux was also the weakest predictor to reach threshold, suggesting mean levels are more important predictors of performance than affect variability indices. Similar to the findings for off-task attention, spin and pulse did not meet threshold and were among the weakest predictors of performance.

**Table 4:** Summary of statistics determining independent variable contributions to regression effects for performance.

Variable	*β*	RWI	RWI %
Pre-change			
Spin	0.010	0.001	0.28
Pulse	−0.030	0.008	2.22
ND flux	0.178	0.010	2.78
NA flux	−0.143	0.024	6.67
PD flux	0.029	0.002	0.56
PA flux	0.005	0.001	0.28
Mean ND	−0.118	0.073	20.28
Mean NA	−0.261	0.076	21.11
Mean PD	−0.015	0.034	9.44
Mean PA	0.366	0.131	36.39
Total	N/A	0.360	100
Post-change			
Spin	−0.003	0.002	5.35
Pulse	0.023	0.005	1.34
ND flux	0.085	0.012	3.21
NA flux	−0.119	0.020	5.35
PD flux	0.058	0.002	0.53
PA flux	−0.030	0.001	0.27
Mean ND	−0.173	0.083	22.19
Mean NA	−0.171	0.062	16.58
Mean PD	−0.011	0.034	9.09
Mean PA	0.390	0.153	40.91
Total	N/A	0.374	100

*β*, standardized regression coefficient in the full model; RWI, relative weight importance; % of variance explained in y by the given index. RWI%, % of variance explained in y by all the indices attributed to the given index. Pre-change refers to performance in the first seven sessions. Post-change refers to performance in the last seven sessions. ND, negative deactivating; NA, negative activating; PD, positive deactivating; PA, positive activating. *N* = 253.

##### 7.1.2.2 Research question 2: dominance

Dominance analysis results across acquisition and adaptation (Table 5) showed complete dominance of mean PA over the other variables, as it explained more unique variance in the regression effect than the other indices across all multiple regression sub-models that include that variable. Additionally, mean PA had the largest beta weight (acquisition: *β* = 0.37, *p* < 0.001; adaptation: *β* = 0.39, *p* < 0.001) in the full model.

**Table 5:** Paired dominance metrics for performance.

	Pre-change performance			Post-change performance		
	Complete	Conditional	General	Complete	Conditional	General
Spin > Pulse	0.5	0.0	0.0	0.5	0.0	0.0
Spin > ND flux	0.5	0.0	0.0	0.5	0.0	0.0
Spin > NA flux	0.0	0.0	0.0	0.0	0.0	0.0
Spin > PD flux	0.5	0.5	0.0	0.5	0.5	0.0
Spin > PA flux	0.5	0.5	0.0	0.5	0.5	1.0
Spin > Mean ND	0.5	0.0	0.0	0.0	0.0	0.0
Spin > Mean NA	0.5	0.0	0.0	0.5	0.0	0.0
Spin > Mean PD	0.5	0.0	0.0	0.5	0.0	0.0
Spin > Mean PA	**0.0**	**0.0**	**0.0**	**0.0**	**0.0**	**0.0**
Pulse > ND flux	0.5	0.0	0.0	0.5	0.0	0.0
Pulse > NA flux	0.0	0.0	0.0	0.0	0.0	0.0
Pulse > PD flux	0.5	0.5	1.0	0.5	0.5	1.0
Pulse > PA flux	0.5	0.5	1.0	0.5	0.5	1.0
Pulse > Mean ND	0.5	0.0	0.0	0.0	0.0	0.0
Pulse > Mean NA	0.5	0.0	0.0	0.5	0.0	0.0
Pulse > Mean PD	0.5	0.5	0.0	0.5	0.5	0.0
Pulse > Mean PA	**0.0**	**0.0**	**0.0**	**0.0**	**0.0**	**0.0**
ND flux > Mean ND	0.5	0.5	0.0	0.0	0.0	0.0
ND flux > Mean NA	0.5	0.0	0.0	0.5	0.0	0.0
ND flux > Mean PD	0.5	0.5	0.0	0.5	0.5	0.0
ND flux > Mean PA	**0.0**	**0.0**	**0.0**	**0.0**	**0.0**	**0.0**
NA flux > ND flux	0.5	0.5	1.0	0.5	1.0	1.0
NA flux > Mean ND	0.5	0.5	0.0	0.0	0.0	0.0
NA flux > Mean NA	0.5	0.0	0.0	0.5	0.0	0.0
NA flux > Mean PD	0.5	0.5	0.0	0.5	0.5	0.0
NA flux > Mean PA	**0.0**	**0.0**	**0.0**	0.0	0.0	0.0
PD flux > Mean ND	0.5	0.0	0.0	0.0	0.0	0.0
PD flux > Mean NA	0.0	0.0	0.0	0.5	0.0	0.0
PD flux > Mean PD	0.5	0.5	0.0	0.5	0.5	0.0
PD flux > Mean PA	**0.0**	**0.0**	**0.0**	**0.0**	**0.0**	**0.0**
PD flux > ND flux	0.5	0.0	0.0	0.5	0.0	0.0
PD flux > NA flux	0.0	0.0	0.0	0.5	0.0	0.0
PA flux > Mean ND	0.5	0.0	0.0	0.0	0.0	0.0
PA flux > Mean NA	0.5	0.0	0.0	0.5	0.0	0.0
PA flux > Mean PD	0.5	0.0	0.0	0.5	0.5	0.0
PA flux > Mean PA	**0.0**	**0.0**	**0.0**	**0.0**	**0.0**	**0.0**
PA flux > ND flux	0.0	0.0	0.0	0.5	0.0	0.0
PA flux > NA flux	0.5	0.5	0.0	0.0	0.0	0.0
PA flux > PD flux	0.5	0.5	1.0	0.5	0.5	0.0
Mean NA > Mean ND	0.5	0.5	0.0	0.5	0.0	0.0
Mean PD > Mean ND	0.0	0.0	0.0	0.0	0.0	0.0
Mean PD > Mean NA	0.5	0.0	0.0	0.5	0.0	0.0
Mean PA > Mean ND	**0.5**	**1.0**	**1.0**	**1.0**	**1.0**	**1.0**
Mean PA > Mean NA	**1.0**	**1.0**	**1.0**	**1.0**	**1.0**	**1.0**
Mean PA > Mean PD	**1.0**	**1.0**	**1.0**	**1.0**	**1.0**	**1.0**

Pre-change refers to the first seven sessions. Post-change refers to the last seven sessions. ND, negative deactivating; NA, negative activating; PD, positive deactivating; PA, positive activating; 1.0, first listed variable is dominant; 0.0, second listed variable is dominant; 0.5, dominance cannot be determined. Bolded font highlights the results for the variable yielding complete dominance overall. *N* = 253.

#### 7.1.3 Suppression ancillary analysis

Although PA flux was below threshold as an important predictor for off-task attention, its negative commonality coefficient (acquisition: common = −0.02; adaptation: common = −0.01) and near-zero correlation (acquisition: *r* = −0.015; adaptation: *r* = 0.048) but larger beta weight (acquisition: *β* = −0.204; adaptation: *β* = −0.145) indicated that PA flux may act as a suppressor or that its inclusion removed irrelevant variance in other predictors (Nathans et al., 2012). Additionally, when examining the average incremental variance explained by PA flux across models of increasing size (e.g., from models with one other predictor to a model with all predictors), PA flux explained more variance as the number of predictors increased, both in acquisition and adaptation. The variance explained by PA flux during acquisition with one other predictor was 0.011, but this increased to 0.022 when all other predictors were included. Similarly, during adaptation, the variance explained began at 0.007 and increased to 0.011. A series of multiple linear regression models revealed that the inclusion of PA flux increased the variance explained by ND flux. Including PA flux increased *R^2^* from 0.115 to 0.162 for acquisition and from 0.166 to 0.201 for adaptation. There was no indication that any of the predictors acted as a suppressor in predicting performance.

#### 7.1.4 Mediation ancillary analysis

Given the results of the relative weights analysis showed mean ND as the dominant predictor of off-task attention and ND flux was the strongest affect variability predictor of off-task attention, we examined the indirect effects of these variables on performance following Hayes and Preacher (2010). We then examined the indirect effect of ND flux on performance, controlling for mean ND. Results showed a statistically significant indirect effect for mean ND (−1.60, [bootstrapped bias corrected CI 99 = −3.12, −0.61], *R^2^* = 0.30), while the indirect effect for ND flux was not significant (−0.77, [bootstrapped bias corrected CI 99 = −2.45, 0.63]).

## 8 Discussion

The premise of the present study is that between-person differences in emotion fluctuations, namely affect variability scores, have potential for extending theory on the roles non-cognitive traits like personality play in behavioral phenomena. However, despite theory suggesting affect variability is distinct from mean levels, empirical investigation has not yet examined the effect of affect variability indices alongside mean levels. Additionally, it is rare for studies to comprehensively consider all indices of affect variability. Thus, the general purpose of this study was to gain a better understanding of how a wide range of affect variability indices compared to mean levels are associated with off-task attention and performance in the context of complex task performance. We disentangled valence and arousal aspects of emotion and showed that mean levels tended to be stronger, more important predictors than affect variability indices for both off-task attention and performance during periods of acquisition and adaptation to unexpected task changes. Specifically, the mean of negative deactivating emotions was the dominant predictor of and positively related to off-task attention, while the mean of positive activating emotions was the dominant predictor of and positively related to performance.

### 8.1 Theoretical and practical implications

Our finding showing negative deactivating emotion mean levels as the dominant predictor of off-task attention with indirect effects undermining performance is consistent with prior research showing how mind wandering occurs when individuals feel concern, worry, and disappointment (Moon et al., 2020). The experience of negative deactivating emotions is associated with task disengagement and reduced motivation (Pekrun, 2011). Additionally, negative affect acts as a distractor that pulls attention off-task (Conway et al., 2013; Rowe and Fitness, 2018), particularly under pressure (Strauss and Allen, 2009). The results for mean positive activating emotions as the dominant predictor of performance is consistent with prior research showing stronger effects for positive activating emotions than positive deactivating emotions for performing fast-paced and dynamic tasks (Jorgensen et al., 2021). Positive activating emotions are associated with processes relevant for task performance, such as engagement, strategy elaboration, and curiosity (Pekrun, 2011; McConnell and Eva, 2012).

The zero-order correlations between mean levels and off-task attention, and mean levels and task performance were consistent with our expectations [i.e., negative (positive) mean levels were positively (inversely) related to off-task attention; positive (negative) mean levels were positively (inversely) related to task performance]. However, in the relative importance analyses the direction of some effects did not match our expectations. Specifically, mean NA and mean PD were negatively related to off-task attention and task performance, respectively. Although additional replicative research is needed, and notwithstanding potential collinearity issues, these unexpected findings may be indicative of the important role of emotional arousal in predicting behavioral outcomes. In regard to attention, emotional activation, regardless of valence, has been associated with greater attention allocation toward relevant information (LeBlanc et al., 2015). For performance, research has found a relationship between deactivating emotions and outcomes that are detrimental for performance gains (i.e., disengagement, lowered learning efforts, and stunted knowledge growth; Pekrun, 2011). These findings support the importance of accounting for both valence and arousal in research examining relationships between affect and behavioral phenomena (Kuppens et al., 2013; Bliss-Moreau et al., 2020).

In general, because mean levels were found to be important and dominant predictors, our findings show it is critical for future research to account for mean levels when examining relationships between affect variability and behavioral outcomes. Mean levels of emotion are important predictors of behavioral outcomes, and our results highlight how there is not enough evidence to ignore the effect of mean levels on outcomes of interest when examining relationships between affect variability indices and outcomes. Conclusions drawn about the role of affect variability indices must be tempered alongside the effects of mean levels. Accordingly, researchers should be cautious of drawing conclusions about the magnitude and meaning of the effects for affect variability indices in research where mean levels are not also examined.

In terms of the effect of affect variability indices after accounting for mean levels, our results showed that flux in ND and NA emotions were important predictors such that higher scores were associated with higher off-task attention and lower performance, respectively. These findings are consistent with prior research showing affect variability, spin in particular, is associated with distress (Beal and Ghandour, 2011), and less engagement at work. Following the change in task demands, NA flux became an important predictor for off-task attention (i.e., NA flux met the *a priori* importance threshold in adaptation, but not in acquisition). This finding supports previous research showing how indices of affect variability are related to poor adjustment to unexpected events (Beal and Ghandour, 2011), such that the effect of NA flux becomes more pronounced when individuals face unexpected changes or increases in task complexity. The process of regulating one’s emotions is mentally fatiguing and cognitively taxing (Grillon et al., 2015). The depletion of resources as a result of this regulation, particularly when learning complex, fast-paced, and dynamic tasks, can lead to activities that are more cognitively restful and thus inhibit engagement and performance (Wehrt et al., 2022). Our results suggest that under fast-paced, dynamic performance contexts, individuals higher in ND and NA flux may have more difficulty regulating their emotions and cognitions toward task accomplishment. Although indices of affect variability were not dominant predictors, the finding that negative flux indices acted as significant predictors suggests that affect variability’s contribution may be relevant to consider in research beyond mean levels and affirms that they are distinct from mean levels (Hardy and Segerstrom, 2017). Our findings echo previous research showing fluctuations in negative affect are inversely related to on-task attention and harmful for performance (Merlo et al., 2018), but they also extend theory by examining the role of arousal and distinguishing ND flux’s association with off-task attention in both acquisition and adaptation from NA flux’s direct relationship with task performance in both acquisition and adaptation coupled with an off-task attention association in adaptation. Moreover, our findings showed positive flux indices were not important to task attention and complex task performance, and in doing so they extend theory by suggesting that affect variability associations with adjustment and performance are driven more by the negative side of the affect circumplex.

The majority of affect variability research has centered around spin and pulse. To our knowledge, this is the first study to compare spin, pulse, and four dimensions (PA, PD, NA, ND) of flux. Thus, the greater importance of flux variables over the traditionally used spin and pulse variables is an important finding. Accordingly, the present study’s findings suggest that flux should be given more empirical attention, and that flux variables that disentangle activation and valence have potential to better explain behavioral outcomes compared to single indices like spin and pulse which may not be able to capture the nuances present within each quadrant of the affect circumplex. For example, a close look at the formula for calculating spin shows that two individuals can receive the same spin scores despite having different patterns across the circumplex (e.g., one varying across PA and ND; the other varying across PD and NA). From a theoretical perspective, utilizing dimensions of flux is more theoretically and computationally parsimonious than pulse or spin. Additionally, nuances in our results signify the importance of investigating emotional arousal (i.e., activation and deactivation) in addition to valence. Historically, affective traits have been categorized as positive or negative, but our findings suggest this view is too coarse and may contribute to mixed findings in the literature regarding the relationship between affect and outcomes of interest (i.e., performance). Our relative importance results for flux were not uniform by valence but varied as a function of activation. This aligns with research that suggests arousal (rather than valence) may be a key determinant of task performance (Baas et al., 2008). Altogether, our findings point to the need for future research to include flux variables that disentangle valence and arousal to expand theory on how affect variability explains variance in behavioral phenomena.

From a practical standpoint, affect variability may be important to consider in selection and performance management contexts (i.e., assessing, measuring, and developing individual capabilities), which involve domains found to be related to affect variability (e.g., task performance, adaptive performance, occupational citizenship behaviors, counterproductive work behaviors; Aguinis, 2009; Smither and London, 2009). In dynamic, fast-paced performance environments where change is to be expected, training and development programs may be leveraged to provide individuals with strategies to mitigate the detrimental effects of affect variability. It is important to note here that fluctuations in emotion are normal, not all fluctuation patterns are detrimental, and average levels are likely more important and practically easier to consider. Although research has found that individuals with higher affect variability experience increased strain and emotional exhaustion due to engaging in greater emotion regulation (Niven et al., 2012; Beal et al., 2013), research on the effectiveness of various emotion regulation strategies for addressing high affect variability is lacking and needed.

### 8.2 Limitations

When interpreting and generalizing the results found here, some limitations should be acknowledged. First, given the college student sample, laboratory context, and performance-based lottery incentive, caution is warranted when generalizing our findings, especially given the fast-paced nature and perceptual-motor demands of the criterion task. Additionally, although not a focus of the present study, it is important to note that that participant interest, emotion regulation, and prior task experience may be factors that contribute to participants’ emotional response, attention, and performance. Indeed, research has found an individual’s ability to regulate their emotions will affect their performance (Niessen and Jimmieson, 2016). Accordingly, future research across a variety of samples and learning and performance contexts that accounts for participant past experience is needed. Second, a common limitation to studies of affect variability repeated here was the measurement of emotions occurred concurrently with the measurement of the outcome variables. Predictive designs that measure affect variability prior to the measurement of outcomes (e.g., task performance) are needed to provide stronger evidence that affect variability scores reflect personality broadly. Additionally, given the practical constraints to measuring affect variability via repeated measures, we believe future research should examine whether one-time, more clinically-oriented measures of emotion reactivity (e.g., Nock et al., 2008; Becerra et al., 2019) are viable substitutes for the repeated-measures approach in affect variability scores or if a commensurate measure of affect variability could be developed. As such, future research should examine the possible tradeoff between administration feasibility and measurement precision.

The use of self-reports of off-task attention is another important limitation. Participants may have struggled to fully monitor how much attention they directed to the task. Some participants may also be hesitant to report they were not paying attention. Eye-tracker technology and other physiological measures, previously used to measure attention and attentional shifts (Maclin et al., 2011), could be used in future research, with the assumption participants are focused on what they visually examine (Duchowski, 2002; Moran et al., 2016). Prior complex skill research has found it possible to utilize other technology, such as electroencephalogram (EEG; Maclin et al., 2011; Bakaoukas et al., 2016) and functional magnetic resonance imaging (fMRI; Prakash et al., 2012), to monitor brain states that reflect attention. As individuals acquire skills, there will be a reduction in the attentional resources devoted to task performance as the execution of performance strategies become automated (Kanfer and Ackerman, 1989; Kanfer et al., 1994; Prakash et al., 2012). As such, neuroimaging and EEG could be better leveraged to capture changes and differences in attention, rather than relying on self-reports, to distinguish whether participants are (1) putting resources toward on-task attention versus (2) activating attentional control areas but failing to succeed in acquiring the skills necessary to perform the task. Physiological techniques may enable a more nuanced examination of the mechanisms tested in the present study.

## 9 Conclusion

This study contributes to our understanding of the relationship between affect variability indices and mean levels of emotions, and their relative importance in predicting complex task performance. Although affect variability indices were not as strong of predictors as mean levels, flux in negative activating and deactivating emotions were nevertheless important predictors, distinct from mean levels. Thus, it may be critical and necessary to measure the dynamic aspects of personality that traditional measures of personality do not capture to extend theory on learning and adaptive performance as well as other behavioral outcomes. We hope the current study motivates future research on affect variability to account for mean levels and comparatively examine affect variability indices to elucidate their respective contributions to learning and performance outcomes and more generally a variety of outcomes associated with adjustment and adaptability.

## Data Availability

The raw data supporting the conclusions of this article will be made available by the authors, without undue reservation.

## Footnotes

^1^We acknowledge that aside from the meta-analyses (Shockley et al., 2012; Fodor and Pintea, 2017) the cited studies methodologies are unique, and results may be context-dependent.

## Author’s note

This research was conducted as part of Maddison N. North’s master’s thesis at the University of Oklahoma under the supervision of Eric Anthony Day. A previous version of part of this manuscript was presented at the 37th Annual Conference of the Society for Industrial and Organizational Psychology.
